# Serum IL-1β, IL-2, and IL-6 in Insulin-Dependent Diabetic Children

**DOI:** 10.1155/MI/2006/59206

**Published:** 2006-02-01

**Authors:** Yasar Dogan, Saadet Akarsu, Bilal Ustundag, Erdal Yilmaz, Metin Kaya Gurgoze

**Affiliations:** ^1^Department of Pediatrics, Medical Faculty, Firat University, 23119 Elazig, Turkey; ^2^Department of Biochemistry, Medical Faculty, Firat University, 23119 Elazig, Turkey

## Abstract

Insulin-dependent diabetes mellitus (IDDM) is a chronic disease characterized by T-cell-dependent autoimmune destruction of the insulin-producing β cells in the pancreatic islets of Langerhans, resulting in an absolute lack of insulin. T cells are activated in response to islet-dominant autoantigens, the result being the development of IDDM. Insulin is one of the islet autoantigens responsible for the activation of T-lymphocyte functions, inflammatory cytokine production, and development of IDDM. The aim of this study was to investigate serum concentrations of interleukin (IL)-1β, IL-2, IL-6, and tumor necrosis factor (TNF)-α in children IDDM. The study population consisted of 27 children with IDDM and 25 healthy controls. Children with IDDM were divided into three subgroups: (1) previously diagnosed patients (long standing IDDM) (*n* : 15), (2) newly diagnosed patients with diabetic ketoacidosis (before treatment) (*n* : 12), and (3) newly diagnosed patients with diabetic ketoacidosis (after treatment for two weeks) (*n* : 12). In all stages of diabetes higher levels of IL-1β and TNF- α and lower levels of IL-2 and IL-6 were detected. Our data about elevated serum IL-1β, TNF- α and decreased IL-2, IL-6 levels in newly diagnosed IDDM patients in comparison with longer standing cases supports an activation of systemic inflammatory process during early phases of IDDM which may be indicative of an ongoing β-cell destruction. Persistence of significant difference between the cases with IDDM monitored for a long time and controls in terms of IL-1β, IL-2, IL-6, and TNF-α supports continuous activation during the late stages of diabetes.

## INTRODUCTION

Recent evidence favors primary role of cellular autoimmunity and its humoral mediators in pathogenesis and following IDDM [1]. IDDM is an autoimmune disease with an inflammatory process directed against the β cells in pancreas [2]. Monocyte and type 1 T-cell-derived cytokines contribute to the pathogenesis of IDDM [3]. Chemokines play a central role in inflammatory processes by regulating leukocyte migration into sites of tissue damage [4]. Cytokines have been proposed as inducers of β-cell damage in human IDDM via the generation of nitric oxide (NO) [5].

The autoantigen insulin is responsible for stimulation in vitro of potentially hazardous memory lymphocytes to produce IL-6 and IL-10 [6]. A T helper 1 (T_H_1) subset of the T cells and their cytokine products (type 1 cytokines: IL-2, interferon (INF)-γ, and tumour necrosis factor (TNF)-β) dominate over an immunoregulatory T_H_2 subset of T cells and their cytokine products (type 2 cytokines: IL-4, IL-5, and IL-13). There is an imbalance between T_H_1 and T_H_2 subsets. This allows type 1 cytokines to initiate a cascade of immune-inflammatory processes in the islet, which includes activating macrophages to produce proinflammatory cytokines. The proinflammatory cytokines, IL-1β, IL-6, and TNF-α, have cytotoxic, cytostatic (inhibits insulin synthesis and secretion), or cytocidal actions to pancreatic islets by inducing NO production [4, 6, 7].

Recent reports suggest that the pancreas participates in TNF-α production during stress, and that the islets are predominantly responsible for such synthesis. IL-1β and TNF-α are important for the β-cell lysis in IDDM, while IL-1 receptor antagonist (IL-1ra) is considered protective by blocking the effects of IL-1 [2]. In vitro TNF-α and IL-1β inhibit insulin release from islet β cells. It appears that the process of autoimmune aggression against β cells, and its effect on insulin release and glucose homeostasis, is a slow and chronic process. However, the production of these cytokines and consequently the degree of β-cell destruction, in a genetically susceptible subject, might be enhanced by several factors including viral infections [8]. In some studies performed with newly diagnosed IDDM patients production of IL-1 was found to be increased significantly when compared with chronic IDDM patients and healthy controls. IL-1ra/IL-1 ratio decreased in patients with ND-IDDM and returned to normal in LS-IDDM group. Circulating concentrations of IL-1ra in LS-IDDM patients have increased. Any change in TNF synthesis was not detected. This data suggests a proinflammatory imbalance in ND-IDDM patients and this may play an important role in β-cell loss [9].

The proinflammatory cytokines IL-1, IL-2, and TNF-α may play important roles alone or in combination in the pathogenesis of IDDM [10]. IL-1β and TNF-α levels can be used as indicators of continuing autoimmune aggression against β cells before the development of extensive β-cell destruction [8, 11]. Circulating IL-6, TNF-α, and CRP have been also determined as markers of the inflammatory response [12].

There is little and conflicting information regarding circulating levels and in vitro production of cytokines in diabetes. Several studies have found an increase in serum IL-6 concentrations [4, 13]; however some studies reported no difference [14] or even decreased [15] IL-6 levels. Results regarding levels and/or production of TNF-α have been inconclusive and are reported to be increased [4, 16], decreased [15], or unchanged [8, 9] as well. The effects of glycemic control on cytokines are consistent in several studies [4, 7], especially in children. The circulating levels of these cytokines in the development of type 1 DM have no conclusion in different studies.

The aim of this study was to investigate serum concentrations of IL-1β, IL-2, IL-6, and TNF-α in children IDDM.

## MATERIALS AND METHODS

The study population consisted of 27 children with IDDM and 25 healthy controls. Children with IDDM were divided into three subgroups: (1) previously diagnosed patients (long standing IDDM) (*n* : 15), (2) newly diagnosed patients with diabetic ketoacidosis (before treatment) (*n* : 12), and (3) newly diagnosed patients with diabetic ketoacidosis (after treatment) (*n* : 12).

After the written informed parental consents were obtained, we collected the serum samples from 27 children with IDDM (12 females, 15 males) at our pediatric diabetes clinics. Subjects with any diabetic complications such as nephropathy, neuropathy or retinopathy, acute or chronic diseases, or oral medication were excluded from this study. All patients were treated with daily regular doses of insulin (1 IU/kg/d). Twenty five children without diabetes (11 females, 14 males) were recruited for the control group. The age range of the subjects was 4–17 years. Baseline features of the groups are presented in Table 1.

The background information of subjects such as age, gender, body weight, body height, daily dose of insulin injection, and disease duration were recorded. Disease duration was defined in this study as the day of initial diagnosis of diabetes to the day of blood collection. Blood samples (five milliliters) were collected using standard venipuncture technique between 9:00 and 11:00 AM. Serum samples were separeted immedately after centrifugation at +4°C, 4000 rpm for 10 minutes and stored at −20°C until analysis.

Percent concentration of HbA1c in whole blood was measured with Roche diagnostics HbA1c kits with autoanalyzer (Cobas Integra 800 Autoanalyzer, Roche Diagnostics, Germany). This assay based on the immunoturbidometric determination of the stable glucose adducct to N-terminal group of the haemaoglobin beta chain. Serum concentrations of cytokines such as IL-1β, IL-2, IL-6, and TNF-α were measured using commercially available enzyme-linked immunosorbent assay kits (ELISA kits, Biosource Int, Calif). All of the serum samples were analyzed in one assay in duplicate with intra- and interassay < 10%. (IL-1β sensitivity < 2 pg/mL, interassay %4.6, intrassay %3.4; IL-2 sensitivity < 0.1 U/mL, interassay %7.5, intrassay %5.7; IL-6 sensitivity < 2 pg/mL, interassay %8.0, intrassay %6.0; and TNF-α sensitivity < 3 pg/mL, interassay %9.9, intrassay %5.2.)

## STATISTICS

Data is presented as mean±SD. Comparison of variables was performed with the general linear model, student *t* test, or Mann-Whitney *U* test when necessary. Case-control differences in nominal data were evaluated with the χ^2^ test. Statistical analysis was performed using SPSS for Windows (SPSS Advanced 7.5, SPSS, Chicago, Ill, 1997). The comparisons of serum cytokine between children with diabetes and healthy were determined by multivariate analysis of variance (MANOVA). A simple linear correlation analysis was processed by Pearson's method to assess the correlation between age, BMI, HbA1c, and cytokine in healthy and diabetic subjects, respectively. A forward stepwise multiple linear regression analysis was done to identify the influence of multiple variables, including age, diabetes, gender, BMI, HbA1c, and cytokine in children with diabetes and healthy, respectively. Statistical significance was assumed at *P* < .05.

## RESULTS

In this study groups mean age and age range were as follows: control group (10.5 ± 1.0 year) (8–13 year), LS-IDDDM group (10.6 ± 2.1 year) (7–15 year), and ND-IDDM group (9.5 ± 1.6 year) (6–13 year). Mean diabetes duration was 2.9 ± 1.0 year in LS-IDDM groups. The present study was carried out to investigate serum concentrations of body mass index (BMI), HbA1c, microalbuminuria, antistreptolysin O (ASO), C-reactive protein (CRP), white blood cell (WBC), and IL-6 and TNF-α in children with IDDM (Tables 1 and 2).

As shown in Table 1, the level of glucose were significantly higher in control groups compared to LS-IDDM and ND-IDDM groups in this study (*P* < .01, *P* < .001). Hba1c levels especially were significantly higher than control group compared to LS-IDDM and ND-IDDM groups. Also there was a significantly increase in the level of microalbuminuria in diabetic groups and the positive correlation was showed between Hba1c level and microalbuminüria in diabetic groups (*r* : 0.71, *P* < .01 and *r* : 0.678, *P* < .01).

In all stages of diabetes higher levels of IL-1β and TNF-α and lower levels of IL-2 and IL-6 were detected. The results are shown in Tables 1 and 2. There was a significantly positive correlation between the levels of IL-1β and IL-6 (*r* : 0.534, *P* < .05) and between the levels of IL-6 and TNF-α (*r* : 0.565, *P* < .05) in LS-IDDM. Also, in ND-IDDM group, a significantly positive correlation between the levels of IL-2 and IL-6 (*r* : 0,698, *P* < .01) and between the levels of IL-2 and IL-6 (*r* : 0.705, *P* < .01) was showed.

## DISCUSSION

Chemokines play a central role in inflammatory processes by regulating leukocyte migration into sites of tissue damage [4]. Cytokines have been proposed as inducers of β-cell damage in human IDDM via the generation of NO [5]. The proinflammatory cytokines IL-6 and TNF-α are common to both T_H_ subsets in humans [4, 10]. TNF-α and IL-6-mediated damage to micro- and macrovascular tissues, altered insulin secretion through direct or through stimulation of free fatty acid production, and altered glucose homeostasis are suggested [17, 18]. IL-6 and TNF-α are adipocyte-secreted factors [16, 17, 19]. TNF-α injection induces an increase in the concentration of plasma triglyceride and very low density lipoproteins [4]. The proinflammatory cytokine production is elevated in diabetes and in cases of elevated lipids. Diabetes induced abnormalities in fatty acid metabolism have the potential to influence macrophage cytokine release inducing upregulation of proinflammatory cytokines [20, 21]. The proinflammatory cytokines IL-1, IL-2, and TNF-α may play important roles alone or in combination in the pathogenesis of IDDM [10]. Circulating IL-6, TNF-α, and CRP have been also determined as markers of the inflammatory response [12].

The IL-2 system which involves IL-2 production, IL-2 receptor expression, and response to IL-2 is associated with autoimmune phenomena. Immunological abnormalities including autoimmune phenomena are believed to contribute to the pathogenesis of IDDM. In IDDM, IL-2 production by CD4-positive T lymphocytes within the IL-2 system is thought to be selectively defective [22]. Deficient production of IL-2 has been reported in IDDM, but its cause has not been elucidated [23, 24]. T lymphocytes are implicated in the pathogenesis of IDDM. IL-2—a T_H_1 lymphocyte-derived cytokine—is at present considered to play an important role in the etiopathogenesis of IDDM. Activated T lymphocytes expressing IL-2 receptors are found at increased levels in the peripheral blood in the prediabetic period, at diagnosis and for several months after the onset of the disease, but their role in the pathogenesis of the disease is not known [25]. In previous studies with variable outcomes, IL-2 levels were found to be increased, decreased [22, 26, 27], or unchanged in patients with IDDM. These differences can be a result of different metabolic status or/and a different stage of the autoimmune process [28].

IL-2 receptors are released in the circulation in response to antigenic or mitogenic stimulation of T lymphocytes. Abnormal serum IL-2 receptor levels have been found in young children with IDDM and prediabetes. This phenomenon is acquired close to disease onset and is unlikely to be an early marker of IDDM [29]. Soluble IL-2 receptor (sIL-2R) levels reflect mononuclear cell activation and are elevated in a variety of autoimmune, neoplastic, and infectious conditions. Several investigators have studied sIL-2R levels in patients with IDDM, but results (low and elevated) have been conflicting [30].

The present study suggests the involvement of IL-2 in the pathogenesis of IDDM. In our study IL-2 levels decreased notably in all groups. Especially in patients monitored for a long time with a diagnosis of IDDM a significant decrease in IL-2 levels is a striking finding (Table 2). These results confirm the presence of an imbalanced cellular immune response in IDDM patients and demonstrate that the IL-2 deficiency is already present at the diagnosis [27]. The decreased IL-2 synthesis is specific for IDDM, not explainable solely as a consequence of poor metabolic control, and thus, might be involved in the pathogenesis of the disease [31]. The low levels of IL-2 might be explained by an abnormal consumption or by the presence of increased sIL-2R levels or by a serum factor which interferes with IL-2 production [32]. These results suggest that in recent onset IDDM, IL-2-receptor-positive circulating T cells require an IL-2 supply [24].

IL-6 might play a significant role in IDDM etiopathogenesis [32]. Diabetic patients have elevated blood levels of IL-6, which is known to increase the inflammation and the development of vascular disease and atherosclerosis [33]. IL-6 levels were found to be decreased statistically significantly in any group of children with IDDM especially in newly diagnosed cases when compared with healthy controls. Even a statistically significant difference was revealed between IL-6 values related to newly diagnosed cases and those found during the posttreatment period (Table 2). In our study IL-6 levels did not reach to those of healthy children even though they rised from their lowest values in newly diagnosed cases to posttreatment values, and increased further in IDDM patients monitored for a long-term. However in some studies IL-6 levels were found to be higher in newly diagnosed cases when compared with those monitored for a long time [4]. The reports of IL-6 abnormal production in patients with IDDM are rare [25].

TNF-α remained at its highest levels in all chronic cases. Even a statistically significant difference existed between newly diagnosed cases and posttreatment group. Higher levels of TNF-α found in every stage of the disease indicate the persistence of activation of systemic immune inflammatory response [4]. In our cases levels of TNF-α increased parallel with the chronicity of the disease. However Erbagci et al [4] found higher levels in newly diagnosed cases. In contrast with our findings, Netea et al [9] revealed that TNF-α concentrations did not change in newly diagnosed cases and chronic IDDM patients.

In this study serum ASO, CRP, WBC, and proinflammatory cytokine concentrations (IL-1β, IL-6) indicated a state of chronic inflammation in all patients with IDDM. Levels of IL-1β and IL-6 in all DM groups were detected to be significantly different from those of healthy controls. TNF-α levels in all DM groups were found to be significantly different from both control and all other groups including posttreatment groups. However Erbagci et al [4] revealed that CRP, IL-6, and TNF-α levels were not different from those of control group.

Changes in interleukin levels are not limited to early stages of the disease but it persist in advanced stages. In some studies these changes were found to be restricted to only the first stage of the disease [31].

In this study we compared patients in different stages of diabetes among themselves and with healthy control group. Our data about elevated serum IL-1β, TNF-α and decreased IL-2, IL-6 levels in newly diagnosed IDDM patients in comparison with longer standing cases supports an activation of systemic inflammatory process during early phases of IDDM which may be indicative of an ongoing β-cell destruction. Persistence of significant difference between the cases with IDDM monitored for a long time and controls in terms of IL-1β, IL-2, IL-6, and TNF-α supports continuous activation during the late stages of diabetes.

## Figures and Tables

**Table 1:** Baseline characteristics of the study groups (mean ± SD).

	Group 1	Group 2	Group 3		

	Control	Long standing IDDM	Newly diagnosed IDDM		
	(C)	(LS-IDDM)	(ND-IDDM)	

			Before treatment	After treatment	*P*-value
			(BT)	(AT)	

*n*	25	15	12	12

Glucose (mg/dL)	84.8 ± 13.8	158.3 ± 21.7	470.9 ± 98.1*a, b*	145.5 ± 24.4	*aP* < .01	ND-IDDM versus C
						ND-IDDM versus
						LS-IDDM
					*bP* < .01	LS-IDDM versus C
					*P* < .001	ND-IDDM
						(BT versus AT)

HbA1c (%)	5.3 ± 0.7	8.0 ± 1.2*b*	9.9 ± 1.4*a*	8.6 ± 1.1	*aP* < .01	ND-IDDM versus C
						ND-IDDM versus
						LS-IDDM
					*bP* < .01	LS-IDDM versus C
					*P* < .05	ND-IDDM
						(BT versus AT)

Microalbuminuria (mg/L)	6.4 ± 1.0	8.1 ± 1.5	10.2 ± 3.8*a*	9.4 ± 2.9	*aP* < .001	ND-IDDM versus C
						ND-IDDM versus
						LS-IDDM

Urea (mg/dL)	21.8 ± 4.2*a*	30.7 ± 6.6*b*	38.1 ± 10.6	31.4 ± 11.8	*aP* < .01	C versus LS-IDDM
						C versus ND-IDDM
					*bP* < .05	ND-IDDM versus
						LS-IDDM
					*P* < .05	ND-IDDM
						(BT versus AT)

Creatinine (mg/dL)	0.5 ± 0.1	0.8 ± 0.1	0.9 ± 0.2*a*	0.8 ± 0.2	*aP* < .01	ND-IDDM versus C
						ND-IDDM versus
						LS-IDDM

Creatinine clearance (ml/dk)	80.5 ± 10.6*a*	56.7 ± 10.9	52.4 ± 13.7	50.1 ± 14.1	*aP* < .01	C versus LS-IDDM
						C versus ND-IDDM

ASO (U/mL)	0.2 ± 0.04*a*	1.5 ± 0.3	2.0 ± 0.5	1.3 ± 0.4*	*aP* < .01	C versus LS-IDDM
						C versus ND-IDDM
					*P* < .05	ND-IDDM
						(BT versus AT)

CRP (mg/L)	0.3 ± 0.04	0.9 ± 0.2	1.3 ± 0.4*a*	0.9 ± 0.3	*aP* < .05	ND-IDDM versus C
						ND- IDDM versus
						LS-IDDM

WBC (/mm^3^)	7745 ± 1412	12126 ± 3611*a*	9294 ± 1647	8548 ± 1593	*aP* < .05	LS-IDDM versus C
						LS-IDDM versus
						ND-IDDM

**Table 2:** Specific parameters of the study groups (mean ± SD).

	Group 1	Group 2	Group 3		

	Control	Long standing IDDM	Newly diagnosed IDDM		
	(C)	(LS-IDDM)	(ND-IDDM)	

			Before treatment	After treatment	*P*-value
			(BT)	(AT)	

*n*	25	15	12	12

IL-1β (pg/mL)	3.8 ± 0.9*a*	12.2 ± 2.1	13.7 ± 2.3	10.7 ± 2.0	*aP* < .01	C versus LS-IDDM
						C versus ND-IDDM
					*P* < .05	ND-IDDM
						(BT versus AT)

IL-2 (U/mL)	7.12 ± 2.03*b*	4.09 ± 1.17	5.78 ± 0.99*a*	5.01 ± 1.93	*aP* < .01	LS-IDDM versus
						ND-IDDM
					*bP* < .001	C versus LS-IDDM

IL-6 (pg/mL)	3.08 ± 0.9*a*	14.7 ± 3.1*b*	9.3 ± 1.8	6.5 ± 1.4	*aP* < .001	C versus LS-IDDM
						C versus ND-IDDM
					*bP* < .01	LS-IDDM versus
						ND-IDDM
					*P* < .001	ND-IDDM
						(BT versus AT)

TNF-α (pg/mL)	5.7 ± 2.3	10.8 ± 13.3*b*	8.0 ± 2.0*a*	6.3 ± 1.5	*aP* < .05	ND-IDDM versus C
						ND-IDDM versus
						LS-IDDM
					*bP* < .05	LS-IDDM versus C
					*P* < .05	ND-IDDM
						(BT versus AT)
